# *TIF1γ*基因启动子区域突变和甲基化与非小细胞肺癌的关系研究

**DOI:** 10.3779/j.issn.1009-3419.2013.05.02

**Published:** 2013-05-20

**Authors:** 龙强 汪, 哲 雷, 霞 刘, 仍允 刘, 洪涛 张

**Affiliations:** 215123 苏州，苏州大学癌症分子遗传学实验室、苏州市癌症分子遗传学重点实验室 Soochow University Laboratory of Cancer Molecular Genetics, Medical college of Soochow University, Suzhou Key Laboratory for Molecular Cancer Genetic, Suzhou 215123, China

**Keywords:** 肺肿瘤, TIF1γ, 甲基化, bisulfite-sequencing PCR, Lung neoplasms, TIF1γ, Methylation, Bisulfite-sequencing PCR (BSP)

## Abstract

**背景与目的:**

TIF1γ（transcription intermediary factor 1 gamma）属于转录中介因子1家族的成员，可干扰TGF-β/Smad信号通路，抑制TGF-β介导的信号传导，且TIF1γ在多种肿瘤细胞的表达减弱或缺失表明TIF1γ在癌症的发生发展过程中起到抑癌的作用。本研究旨在探索TIF1γ在非小细胞肺癌（non-small cell lung cancer, NSCLC）细胞与组织中的表达差异以确定TIF1γ与肺癌发生关系以及肺癌细胞中TIF1γ表达调控的潜在机制。

**方法:**

选取13例NSCLC患者癌组织以及对应的癌旁组织样本，培养正常支气管上皮细胞HBE和NSCLC细胞株：A549和95C。运用Real-time PCR和Western blot检测细胞与组织中*TIF1γ*基因的表达，并用ImageJ灰度扫描软件计算TIF1γ的相对表达量。通过DNA测序的方法检测*TIF1γ*基因启动子区域突变情况，并采用Bisul?te-sequencing PCR（BSP）克隆测序方法检测*TIF1γ*基因启动子区域甲基化情况。

**结果:**

相对于HBE，TIF1γ mRNA和蛋白在A549和95C中明显下调（*P* < 0.05），13对组织样本中，9个（69.2%）癌旁组织样本里TIF1γ mRNA的表达量高于癌组织样本（*P* < 0.05）。突变检测表明*TIF1γ*基因启动子区-287﹣-5在细胞株中没有突变发生。经BSP克隆测序方法分析发现在*TIF1γ*基因启动子-287﹣-5区域里存在5个可被甲基化的CpG位点（-214、-128、-124、-65和-55）。但这些CpG位点的甲基化频率在NSCLC细胞株中相比HBE没有明显差异。

**结论:**

TIF1γ在NSCLC的发生中可能起抑癌的作用，*TIF1γ*基因启动子区域-287﹣-5在正常细胞和NSCLC细胞中都没有发生突变，但在-287﹣-5区域中存在5个可被甲基化的CpG位点。

## Abstract (en)

**Background and objective:**

TIF1γ (transcription intermediary factor 1 gamma), which belongs to transcription intermediary factor 1 family, is an inhibitor of the TGF-β/Smad signaling pathway and could inhibit the signal transduction mediated by TGF-β. The deficiency of TIF1γ expression in a variety of tumor cells suggests that TIF1γ may play as a tumor suppressor gene in cancer development. The aim of this study is to confirm the relationship between TIF1γ and non-small cell lung cancer (NSCLC) through exploring the expression of TIF1γ in NSCLC cells and tissues, and investigate the regulation mechanism of TIF1γ expression in NSCLC cells.

**Methods:**

Thirteen NSCLC and the paired corresponding para-cancerous lung tissue samples and three cell lines (a normal bronchial epithelial cell line HBE and two NSCLC cell lines A549 and 95C) were selected. The quantitative real-time PCR and Western blotting were used to determine the expression of TIF1γ, and ImageJ was used to evaluate the relative expression of TIF1γ. Direct sequencing was performed to detect mutations within the promoter region of the *TIF1γ* gene and then Bisul?te-sequencing PCR (BSP) and cloning sequencing were carried out to test the methylation status of the promoter region of *TIF1γ* gene in the selected cell lines.

**Results:**

Both mRNA and protein expression of TIF1γ were found significantly decreased in A549 and 95C compared with those in HBE (*P* < 0.05). And in 9 pairs (69.2%) of tissues among the 13 pairs, the mRNA expression of *TIF1γ* gene was lower in the cancer tissues than that in the paired paracancerous lung tissues (*P* < 0.05). No abnormal mutation was found in the -287 to -5 region of the promoter of *TIF1γ* gene in the three cell lines. Moreover, five CpG sites (including -214 bp, -128 bp, -124 bp, -65 bp and -55 bp) were detected in the promoter of *TIF1γ* gene by using BSP, and the methylation profiles in these CpG sites showed similar pattern between NSCLC cells and HBE cells.

**Conclusion:**

TIF1γ may play a tumor suppressor role in the progression of NSCLC. No mutation was found in the -287- -5 region of the promoter of *TIF1γ* gene in a normal bronchial epithelial cell line and two NSCLC cell lines. But there are five CpG sites which can be methylated located in this region.

肺癌包括非小细胞肺癌（non-small cell lung cancer, NSCLC）和小细胞肺癌（small cell lung cancer, SCLC），是世界上发病率和死亡率极高的恶性肿瘤之一，其中NSCLC约占肺癌总数的80%-85%。每年的癌症死亡患者中，NSCLC因预后差、治疗效果不明显、存活率低等原因而占据首位^[1]^。近30年来，尽管在肺癌治疗方面有了一定的进展，但由于肺癌生物学特性十分复杂，恶性化程度高，肺癌患者的生存率仍然不容乐观。

TIF1γ（transcription intermediary factor 1 gamma）是近年来新发现的一种新的肿瘤抑制因子，是一种普遍存在的核蛋白属于转录中介因子1家族的成员，因TIF1γ可作为转录辅助因子阻断TGF-β/Smad信号通路而受到广泛的关注^[2, 3]^。TIF1γ的典型特征是在N末端有三个结构域，包括一个RING、两个B-box（一个Ⅰ型B-box、一个Ⅱ型B-box）和一个coiled-coil结构域；在C末端有一个PHD结构域和一个bromo结构域^[4, 5]^。

TGF-β（transforming growth factor β）是一类具有多功能生物活性的细胞因子，能够通过调节细胞的增殖、分化、凋亡、粘附、侵袭和微环境来调节机体的生理过程^[6]^。典型的TGF-β信号通路首先是TGF-β与Ⅱ型TGF-β受体（TGFβRII）结合，然后与Ⅰ型TGF-β受体（TGFβRI）形成复合物使之激活，TGFβRI磷酸化激活R-Smad（Smad1、2、3、5、8）成员，R-Smad再与Co-Smad（Smad4）结合形成复合物转入细胞核内调控靶基因的转录^[7]^。对TIF1γ的功能研究表明TIF1γ在体内主要是通过影响TGF-β信号通路的传导发挥作用，TIF1γ可通过其C末端的PHD结构域，经泛素化途径降解Smad4在细胞内的表达或者竞争性的与Smad4结合从而抑制R-Smad与Smad4的结合，使TGF-β信号通路传导受阻，影响TGF-β介导的生物学过程^[8, 9]^。

研究^[10]^显示，TIF1γ在多种肿瘤细胞表达减弱和缺失，TIF1γ可与TIF1α或TIF1α和TIF1β形成调节复合物抑制小鼠肝癌的发生，TIF1γ的表达减弱可促进小鼠肝癌的发生。同样，在TGF-β诱导的上皮间质转化（epithelial-to-mesenchymal transition, EMT）过程中，TIF1γ可拮抗性地与Smad4作用，TIF1γ的表达缺失可促进TGF-β诱导的EMT^[11]^。在小鼠胰腺癌的发生过程中，TIF1γ也可通过非依赖于Smad4的途径抑制小鼠胰腺癌的发生^[12]^。这些实验表明TIF1γ在癌症的发生发展过程中起到抑癌基因的作用。

然而，有关TIF1γ在肺癌细胞中是否异常表达、肺癌细胞中TIF1γ表达调控机制以及TIF1γ与肺癌发生关系的研究仍未见报道。本研究采用Real-time PCR和Western blot方法检测13对NSCLC组织和2株NSCLC细胞株（A549和95C）以及1株正常人支气管上皮细胞（human bronchial epithelial cell, HBE）中TIF1γ mRNA和蛋白的表达，并用DNA测序和Bisulﬁte-sequencing PCR（BSP）克隆测序方法分析HBE、A549以及95C中*TIF1γ*基因启动子区突变和甲基化情况。

## 1 材料与方法

### 1.1 标本及细胞

13例NSCLC组织样本为苏州大学附属第一医院2011年1月-2012年1月新鲜手术切除样本，患者手术前均未接受放、化疗。肿瘤组织均取自肿瘤中心非坏死部位，癌旁组织取自肿瘤旁约5 cm正常肺组织，所有样本均在医院病理科经H & E染色以确定肺癌样本含有足够的癌细胞（通常癌细胞含量 > 70%），并且经过仔细病理检验以确诊和确定肿瘤的组织分化类型。组织标本切除后30 min内入液氮保存。实验用3株细胞分别是：HBE是正常人支气管上皮细胞，A549、95C分别是具有转移倾向的和低转移的NSCLC细胞株。

### 1.2 荧光实时定量PCR（Real-time PCR）检测*TIF1γ*基因的表达

采用Trizole一步法进行组织和细胞总RNA的提取，采用nanodrop2000分光光度计和琼脂糖凝胶电泳检测总RNA的浓度、纯度和完整性。采用逆转录试剂盒M-MLV first strand kit（Invitrogen）逆转录合成cDNA。采用Platinum SYBR Green qPCR SuperMix（Invitrogen）荧光实时定量试剂盒检测基因mRNA表达量。荧光实时定量PCR的循环条件为：95 ℃预变性10 min，进入40个循环，95 ℃变性30 s，62 ℃退火30 s，72 ℃延伸1 min，并收集荧光信号，72℃延伸10 min，终止反应。TIF1γ以及内参引物见表 1。

**1:** 实时定量PCR引物 Primers used for real-time PCR

Gene	Primer sequence	Product length (bp)
*TIF1γ*	FP: AGCAACGGCGACATCCA RP: AGCAACGGCGACATCCA	61
*β-actin*	FP: GGCGGCACCACCATGTACCCT RP: AGGGGCCGGACTCGTCATACT	202
*GAPDH*	FP: TGCACCACCAACTGCTTAGC RP: GGCATGGACTGTGGTCATGAG	87

### 1.3 Western blot法检测TIF1γ蛋白表达

所有细胞和组织总蛋白的提取步骤严格按照蛋白提取试剂盒ProteoJET^TM^ Mammalian Cell Lysis Reagent（Fermentas）说明书操作，提取中加入蛋白酶抑制剂和磷酸酶抑制剂（Sigma）防止蛋白降解，之后用Thermo公司nanodrop2000分光光度计检测蛋白的浓度和纯度。蛋白经SDS-PAGE变性分离、湿法转膜、封闭、孵育一抗、洗脱、孵育二抗、洗脱、ECL显色、压片后最终检测出TIF1γ蛋白在正常人支气管上皮细胞HBE和NSCLC细胞A549和95C中的表达情况。

### 1.4 DNA的抽提

采用酚-氯仿抽提法抽提DNA。提取出来的DNA用nanodrop2000分光光度计测定浓度和纯度。

### 1.5 重亚硫酸盐修饰基因组DNA

采用EpiTect Bisulfite Kit试剂盒（QIAGEN）对基因组DNA进行重亚硫酸盐修饰。

### 1.6 重亚硫酸盐测序PCR（Bisulfite Sequencing PCR, BSP）法检测*TIF1γ*基因启动子区甲基化

经修饰的基因组DNA进行PCR扩增所需片段，引物FP：TTTAGTTAAAGGTTATTTAGCGTTA；RP：AACAAAAACGACAACCGAAAA。PCR反应体系25 μL如下：2×MightyAmp Buffer 12.5 μL；Primer F 1.0 μL；Primer R 1.0 μL；MightyAmp聚合酶0.5 μL；ddH_2_O 8.0 μL；DNA模板2.0 μL。PCR循环条件为：98 ℃预变性2 min，进入35个循环，98 ℃变性10 s，60 ℃退火15 s，68 ℃延伸1 min，PCR产物经琼脂糖凝胶电泳分离，经溴化乙锭染色后在紫外灯下观察。

### 1.7 PCR产物的T载体克隆

通过PCR扩增目的基因片断，将获得的目的片断通过TA克隆的方法重组到T载体中，经转化后将连接有目的片段的质粒抽提，用BigDye Terminator Cycle Sequencing Reaction Kit在ABI 377自动序列分析仪上测序。

### 1.8 基因突变分析

PCR方法扩增*TIF1γ*基因启动子区上游-287﹣-5序列，将获得的目的DNA片段直接测序分析。

### 1.9 统计分析

所有统计分析均通过SPSS v17.0统计软件完成。利用单因素方差分析统计多组数据间的差异。*P* < 0.05认为具有统计学差异。

## 2 结果

### 2.1 TIF1γ mRNA和蛋白表达情况

TIF1γ在正常支气管上皮细胞HBE中的mRNA表达高于A549和95C这两种NSCLC细胞株（*P* < 0.001）（图 1A）。Western blot检测显示TIF1γ蛋白在各株细胞中的表达与mRNA的表达相一致（图 1B、C）。对13例NSCLC癌与癌旁组织样本中TIF1γ的mRNA表达进行检测，发现9个癌旁组织样本中TIF1γ的mRNA表达高于癌组织样本，说明TIF1γ在NSCLC的发生发展中可能起到抑癌作用。

**1:**
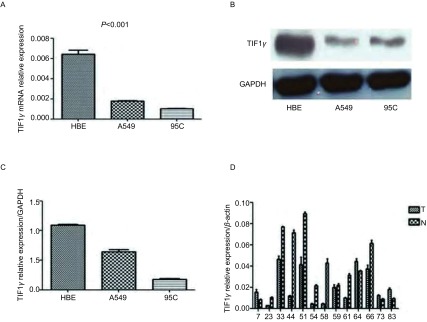
NSCLC细胞株和患者中TIF1γ的表达。A：实时定量PCR法检测HBE、A549和95C细胞中TIF1γ mRNA水平的表达；B：Western blot检测HBE、A549和95C细胞中TIF1γ蛋白的表达；C：用ImageJ灰度扫描软件计算TIF1γ和GAPDH的表达，并用TIF1γ/GAPDH的值作为TIF1γ的相对表达量；D：实时定量PCR法检测13对非小细胞肺癌组织与癌旁组织中TIF1γ mRNA的表达，每对癌组织与癌旁组织均取自同一个体。 Expression of TIF1γ in NSCLC cell lines and NSCLC patients. A: The mRNA levels of TIF1γ and GAPDH in HBE, A549 and 95C were determined by quantitative RT-PCR; B: Representative Western blot of TIF1γ protein levels in HBE, A549 and 95C NSCLC cells; C: Relative estimation for TIF1γ expression in cell lines. ImageJ was used to evaluate the gray value of TIF1γ and GAPDH, and the ratio of TIF1γ/GAPDH was used as relative expression; D: Reletive expression of TIF1γ mRNA in human NSCLC tissues (T) and their paired normal lung tissue (N), each pair obtained from the same patient. NSCLC: non-small cell lung cancer.

### 2.2 *TIF1γ*基因启动子区甲基化情况

在针对小鼠和人的慢性单核型细胞白血病患者的研究中发现，甲基化可以影响TIF1γ蛋白在小鼠和人的慢性单核型细胞白血病患者细胞中的表达^[13]^。图 2A显示了TIF1γ启动子-5﹣-287区域所有CpG位点，通过BSP测序发现该序列中存在5个可被甲基化修饰的CpG位点：-214、-128、-124、-65、-55（图 2B）。并单独计算每个位点甲基化频率（图 2C）。尽管这5个CpG位点单独的甲基化频率在HBE、A549和95C细胞中并未见明显差异，但我们并不能排除其它细胞可通过这5个CpG位点的甲基化调控TIF1γ的表达。

**2:**
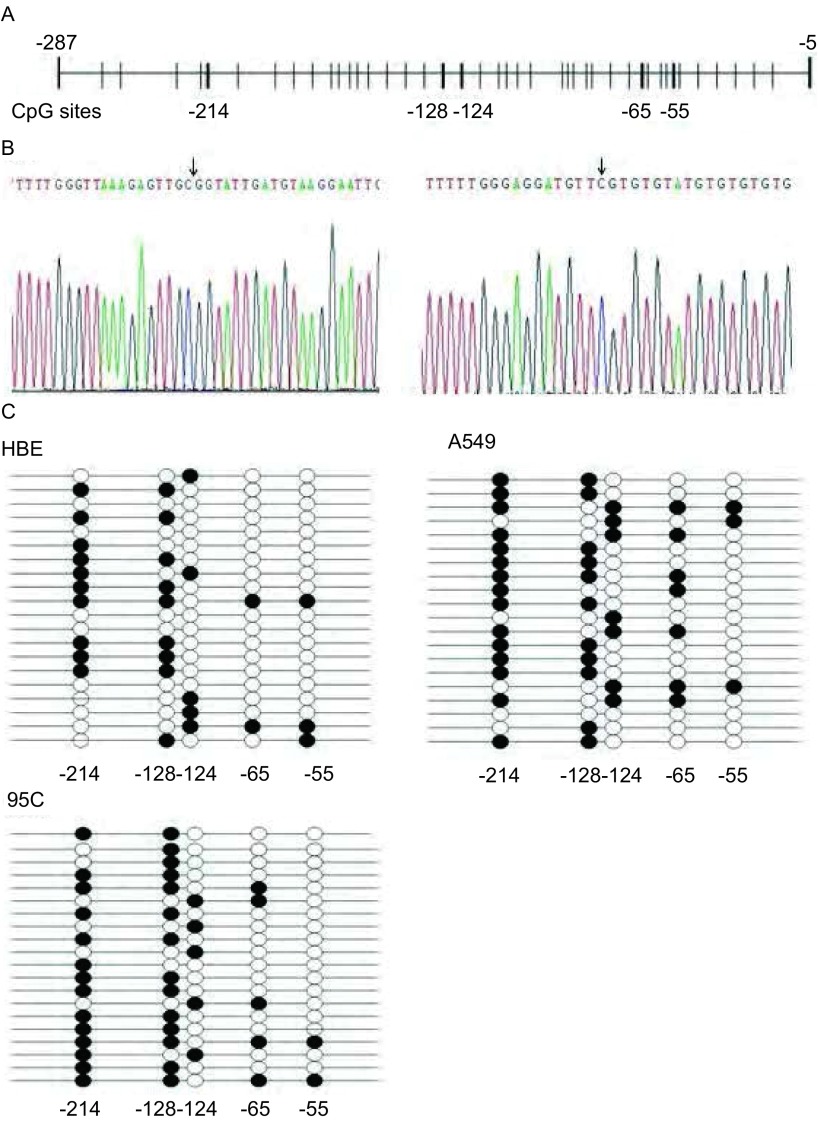
*TIF1γ*基因启动子甲基化状态。A：*TIF1γ*基因-287至-5区域CpG位点分布图；B：*TIF1γ*基因-287至-5区域BSP测序代表图。黑色箭头：甲基化的CpG位点；C：HBE、A549和95C细胞中*TIF1γ*基因启动子-287至-5区域经BSP测序甲基化的CpG位点结果；●：甲基化的CpG位点；○：非甲基化的CpG位点。 Methylation status of TIF1γ promoter. A: The distribution of CpG sites in the -287 to -5 region of TIF1γ promoter; B: Schematic representative of BSP for the promoter region from site -287 to -5 bp of the *TIF1γ* gene. Black arrow: methylated CpG sites; C: Results of methylated CpG sites in the -287 to -5 region of TIF1γ promoter in HBE, A549 and 95C cells by BSP-based sequencing; ●: methylated CpG site; ○: Unmethylated CpG site.

### 2.3 *TIF1γ*基因启动子区突变分析

通过PCR法扩增TIF1γ基因启动子区DNA序列，然后直接测序。经检测，未见突变发生（图 3）。

**3:**
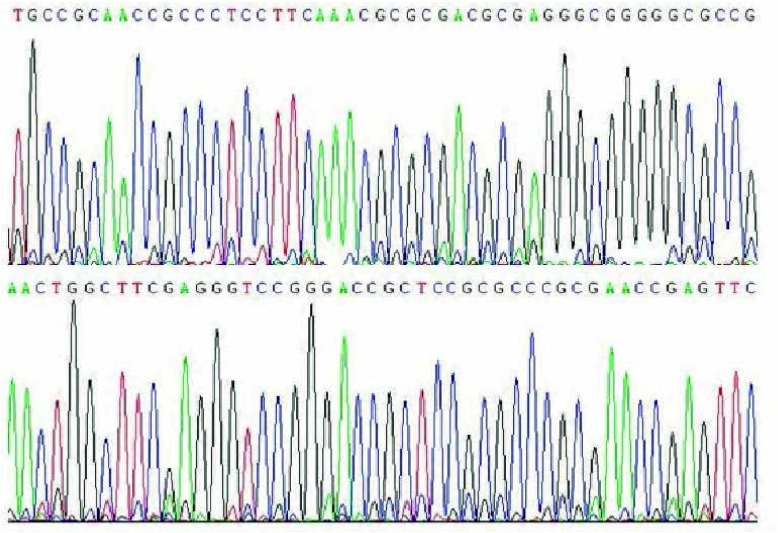
HBE、A549和95C细胞中*TIF1γ*基因启动子-287至-5区域直接测序图。 Schematic representative of direct sequencing for the promoter region from site -287 bp to -5 bp of the *TIF1γ* gene in HBE, A549 and 95C cells.

## 3 讨论

研究^[13]^表明，在人和小鼠的慢性白血病发生过程中，TIF1γ可作为转录辅助因子抑制TGF-β/Smad信号通路，从而抑制癌变。TGF-β信号通路在癌症的发生过程中起着重要的作用，在正常细胞中TGF-β作为癌症抑制因子通过抑制细胞的增殖和诱导细胞的凋亡阻止癌症的进程，然而在晚期癌症细胞中TGF-β可作为癌症促进因子促进癌细胞的侵入和血管的生成^[14]^。TIF1γ与R-Smad竞争结合Smad4或者通过泛素化途径降解Smad4都可使晚期促癌的TGF-β信号减弱，从而起抑癌的作用^[8, 9]^。本研究通过检测TIF1γ在13对癌与癌旁组织中的mRNA表达以及检测TIF1γ在HBE和A549、95C细胞中的mRNA和蛋白表达预测TIF1γ与NSCLC发生的关系，结果表明TIF1γ在NSCLC的发生中可能发挥抑癌的作用。

DNA甲基化作为基因转录调节的一种方式可通过影响癌基因或者抑癌基因的表达从而影响癌症的发生。例如许多抑癌基因原本非甲基化的CpG岛的超甲基化使得基因表达失活，从而诱发肿瘤^[15]^。因此研究抑癌基因的异常甲基化对于肿瘤的早期诊断具有重要的指导意义。本实验首次研究了*TIF1γ*基因启动子区域的甲基化与NSCLC之间的关系。在对*TIF1γ*基因启动子-287﹣-5区域的突变检测表明在各细胞中-287﹣-5区域并没有发生突变，但经BSP分析*TIF1γ*基因启动子-287﹣-5区域甲基化情况后，我们发现在-287﹣-5区域里存在5个可被甲基化的CpG位点（-214、-128、-124、-65和-55），考虑到转录因子结合位点上的CpG发生甲基化会阻碍转录因子的正确结合，从而影响基因转录，所以我们检测了这5个CpG位点在HBE、A549和95C细胞中的甲基化频率。尽管这5个CpG位点甲基化频率在HBE、A549和95C细胞中并未见明显差异，但这5个CpG位点的存在可以为其它细胞调控TIF1γ的表达提供可能途径，因此其它细胞可能会通过*TIF1γ*基因启动子区甲基化调控TIF1γ蛋白的表达。

TIF1γ影响TGF-β介导的生物学过程除了可通过依赖于Smad4的信号通路外还可以通过非依赖于Smad4的信号通路。在造血干细胞的生长分化过程中，TGF-β作为生长抑制因子可通过TGF-β/Smad通路抑制造血干细胞的生长，该过程包括p21蛋白的诱导生成。然而，TGF-β同样可作为细胞分化诱导因子通过下游的R-Smad与TIF1γ形成的复合物诱导造血干细胞的分化^[8]^。

目前为止，TIF1γ通过何种分子机制影响NSCLC的发生发展仍旧不是非常清楚。本研究我们分析了*TIF1γ*基因启动子-287﹣-5区域里的突变和甲基化情况对TIF1γ蛋白表达的影响，而对于其它可能机制并未研究。TIF1γ在NSCLC细胞和组织中的缺失可能涉及到其它表观遗传学上的改变如组蛋白的去乙酰化或其它遗传结构的改变，这有待进一步深入研究。

综上所述，TIF1γ的表达减弱在NSCLC的发生发展过程中可能起着重要的作用，*TIF1γ*基因启动子-287﹣-5区域存在5个可被甲基化的CpG位点，尽管这些CpG位点的甲基化频率在NSCLC细胞株中相比HBE没有明显差异，但并不排除其它细胞可以通过这5个CpG位点的甲基化调控TIF1γ的表达。由于肺癌生物学特性非常复杂，恶性程度高，而且预后的效果差，因此我们必须寻找更加精确、有效的分子标志物，以期更早的发现肺癌，TIF1γ或许可以为NSCLC的治疗提供一个潜在的靶位点。
